# A Misdiagnosed Case of Endoleak Type-III Caused by Inadvertent Bilateral Limb Insertion in the Same Limb Gate of the Ovation Endograft

**DOI:** 10.1055/s-0042-1743197

**Published:** 2022-05-31

**Authors:** Efstratios Georgakarakos, Andreas Koutsoumpelis, Panagiotis Kostoglou, Kalliopi-Maria Tasopoulou, Christos Argyriou

**Affiliations:** 1 Department of Vascular Surgery, “Democritus” University of Thrace, University Hospital of Alexandroupolis, Alexandroupolis, Greece; 2 First University Surgical Department, University Hospital of Alexandropoulis, Democritus University of Thrace, Alexandroupolis, Greece

**Keywords:** endoleak, Ovation, endovascular aneurysm repair, Amplatz, occluding plug

## Abstract

We describe an infrequent case of endoleak Type-III due to an unrecognized, inadvertent bilateral limb deployment into the same limb gate of the Ovation aortic endograft, accompanied by thrombosis and acute ischemia. The following computed tomography angiography revealed the open limb gate with the characteristic of radio-opaque polymer in the sealing rings. Intraoperative angiographies via the brachial route identified the open limb gate and facilitated the successful use of an occluding plug to manage the Type-III endoleak.

## Introduction

The Ovation trimodular endograft is based on a pair of polymer-filled sealing rings to ensure optimal sealing on the infrarenal neck of abdominal aortic aneurysms (AAAs), accommodating even to challenging neck anatomies. 1 Yet, absence of integrated radiopaque markers onto the limb gates of this endograft makes their visualization difficult, rendering sometimes the catheterization of the contralateral limb challenging, especially in cases of anteroposterior limb orientation. 2 Therefore, our case presented below illustrates a relevant problem, the diagnostic pitfalls, and the successful management. Informed consent was obtained from the patient for presentation of the case and relevant images.

## Case Presentation

A 62-year-old male presented with an infrarenal AAA of 51 mm. The treatment chosen for the patient was endovascular repair with the use of the Ovation endograft (Endologix, Irvine, CA) and a main-body 29 mm with iliac limbs of 14 × 16 mm and 14 × 14 mm for the right and left side, respectively. After deployment of the endograft and during polymer filling of the inflated rings, the impression of inadvertent anteroposterior position of the limb gates was given. Under left anterior oblique view, the contralateral limb catheterization was assumed to take place uneventfully, confirmed with the classic tests to ensure proper position. 3 In completion angiography the impression of either a Type-Ia or -II endoleak was mistakenly given. The patient was released on the second postoperative day but admitted urgently to the hospital after 1 month due to acute ischemia of the left lower limb.

**Fig. 1:**
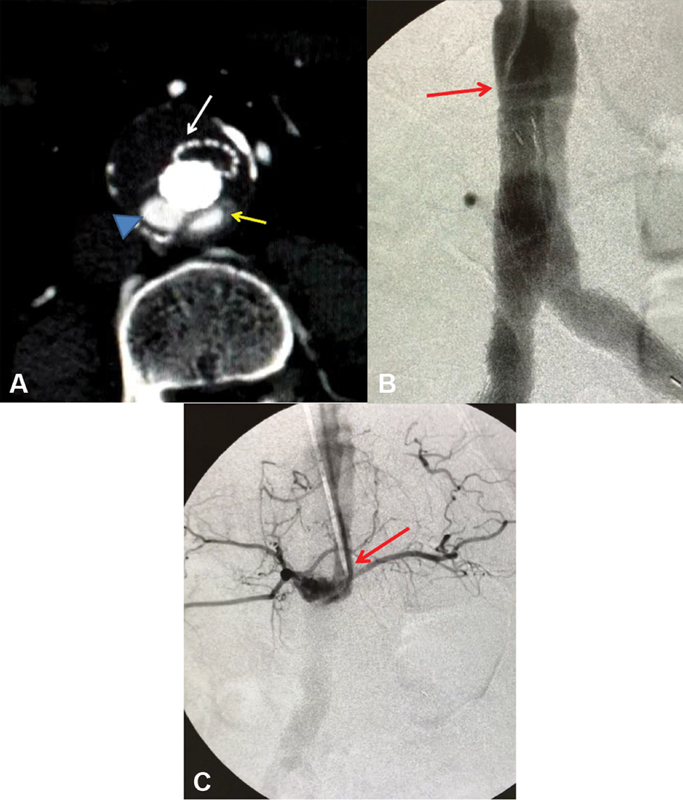
( **A** ) Computed tomographic angiography conducted during the emergent readmission of the patient due to acute ischemia of the left limb. Occlusion and collapse of the left iliac limb of the endograft is shown (white arrow) while buildup of the contrast agent (arrowhead) reveals an endoleak. Careful inspection of the opacification on the left reveals (yellow arrow) a rim encircling the former, corresponding to the radiopaque filling polymer within the Ovation sealing ring, thus identifying the endoleak as Type-III. ( **B** ) Τhe brachial angiographic catheter advanced and placed on the right (arrow) of the endograft shows both iliac limbs normally perfused. No endoleak is visualized. ( **C** ) Withdrawal and placement of the catheter on the left of the main body (arrow) unveils the Type-III endoleak, perfusing exclusively the aneurysm sac; note the lumbar arteries network.

A computed tomography angiography (CTA) was conducted showing placement of both iliac limbs within the same limb gate on the right accompanied by collapse and thrombosis of the left iliac limb ( Fig. 1A , white arrow). While pooling of the contrast agent confirming the endoleak was prominent ( Fig. 1A , arrowhead), careful inspection revealed a gray halo encircling the second dye sequestration on the right ( Fig. 1A , yellow arrow), corresponding to the radiopaque solidified polymer within the lowest sealing ring of the left limb gate seen open. Therefore, the endoleak was now identified as Type-III, attributed to inadvertent bilateral iliac limb insertion into the same (right) gate without cannulation of the contralateral gate.

To restore directly perfusion of the left limb, the patient was subjected immediately to successful open thrombectomy of the left limb, followed by placement of balloon-expandable stents (9 × 36 mm, Valeo, BARD, Peripheral Vascular, Tempe, AZ) at the top end of both iliac limbs with restoration of distal palpable pulses on the left. Accordingly, a 16-mm nitinol-meshed occlusion plug (Amplatzer device; St. Jude Medical, Plymouth, MI) was ordered to treat the endoleak.

**Fig. 2:**
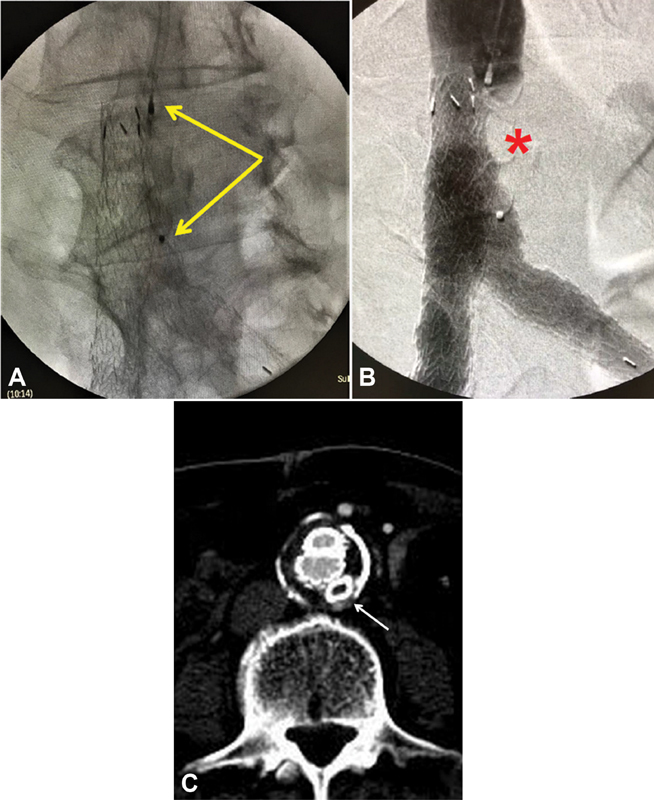
( **A** ) Accurate placement of the Amplatzer plug within the open left iliac limb. The arrows correspond to the markers of the plug. Note the anteroposterior position of both iliac limbs. ( **B** ) Successful occlusion of the left iliac limb (asterisk). ( **C** ) Follow-up computed tomography angiography at 6 months confirms patency of both iliac limbs. The arrow depicts the occluding plug (no contrast).

After a few days, open vascular access was achieved through the left brachial approach. An angiographic catheter was advanced to the endograft's main body and placed within the right limb gate ( Fig. 1B , arrow) perfusing sufficiently both iliac limbs, without revealing any endoleak. On the contrary, slight withdrawal of the catheter and selective placement via the open left limb gate ( Fig. 1C , arrow) enables protrusion in the AAA sac and exclusive visualization of the lumbar arteries, documenting the Type-III endoleak. Accordingly, releasing the occluding plug in the proper position led to complete elimination of the endoleak ( Fig. 2A, B ). The 12-month follow-up CTA confirmed complete sealing of the endoleak and patency of both iliac limbs ( Fig. 2C ).

## Discussion

Our article presents a challenging case of identifying correctly and catheterizing the contralateral limb gate of an endograft for treatment of AAA. Moreover, it underscores the diagnostic difficulty and uncertainty that a nonexperienced radiologist or interventionist may encounter to identify the proper type of the emerging endoleak.

Since both iliac limbs in our case were adequately perfused at the end of the implantation, the retrograde angiographic control via the iliac route proved inappropriate to identify the intrasac leak, due to the anteroposterior position of the endograft allowing no splay of the limb gates. Another important feature for identifying properly the endoleak type was the awareness of the radio-opacity of the filling polymer within the sealing rings of Ovation ( Fig. 1A ). Apart from the opacity of the sealing rings, another unique feature of the Ovation endograft is the lack of nitinol endoskeleton in its main body which can be often perceived as disengagement between the modular parts of the endograft. 4

Notably, the continuous education of radiologists should keep up with the advent of new endografts, especially those involving radiopaque polymer agents associated with consequent findings that could be missed or misinterpreted. 4 5 In doubtful cases, anterograde angiography via the brachial route is recommended, because this maneuver provides ease and accuracy to selective placement of the angiographic catheter to inspect potential sites of endoleaks and delineate diagnostic dilemmas. 6 Furthermore, the anterograde route can facilitate and support the fast advancement, positioning, and very accurate deployment of the occluding plug; the Amplatzer plug is an ideal agent to occlude large endoleaks and can be advanced from the brachial route via an 8 F × 65 cm guiding catheter. 7 8

It should be mentioned that the aforementioned difficulty presented with the particular endograft has been properly addressed recently with the incorporation of a crossover lumen in the endograft, facilitating reliable contralateral gate access in the current Ovation iX design, which should be used in any case of questionable limb cannulation. Yet, such intraoperative challenges with respect to visualization and catheterization of the contralateral limb can be encountered with all other endografts. Therefore, in such cases we strongly advocate anterograde selective angiography and catheterization via the brachial access without delay, as well as multiple oblique projections of the C-arm to ensure the optimal visualization during catheterization.

In cases of ambiguous catheterization of the endograft's contralateral limb gate during endovascular treatment of AAA, high level of suspicion, multiple projections, and anterograde selective angiography via the brachial route should spare intraoperative time and avoid inadvertent failures. Availability of proper embolization materials render the management of endoleaks Type-III quick and effective, while the acknowledgment of each new endograft's special, unique features precludes misdiagnosis and dictates proper management.

The educational value of this report underscores the comparative advantage of the anterograde angiography via the brachial route in ambiguous diagnostic cases and marks the need for continuous education of physicians on the unique, specific features and structure of new endografts to avoid delay in detection and misdiagnosis of associated complications.

## References

[1] Georgakarakos E Ioannou C V Georgiadis G S The ovation abdominal stent graft for the treatment of abdominal aortic aneurysms: current evidence and future perspectives Expert Rev Med Devices 2016 13 03 253 262 26822951 10.1586/17434440.2016.1147949

[2] Georgakarakos E Trellopoulos G Ioannou C V Tsetis D Technical challenges encountered during deployment of the ovation abdominal aortic stent-graft system J Endovasc Ther 2014 21 02 333 338 24754296 10.1583/13-4515MR.1

[3] Trellopoulos G Georgiadis G S Nikolopoulos E S Lazarides M K Current tips for ensuring successful transfemoral short limb cannulation in modular aortic endografts: a new method for incorporation in practice Perspect Vasc Surg Endovasc Ther 2009 21 04 232 236 20628094 10.1177/1531003510365002

[4] Cannavale A Lucatelli P Corona M Current assessment and management of endoleaks after advanced EVAR: new devices, new endoleaks? Expert Rev Cardiovasc Ther 2020 18 08 465 473 32634069 10.1080/14779072.2020.1792294

[5] Chaudhuri A “The Ovation Trap”: when an endoleak is not an endoleak Eur J Vasc Endovasc Surg 2020 60 02 292 32586709 10.1016/j.ejvs.2020.04.003

[6] Georgakarakos E Ioannou C V Kontopodis N Tsetis D A case of difficult catheterization of the contralateral limb of the Ovation Abdominal Stent Graft System in challenging aortoiliac anatomy, facilitated through the brachial access: a word of caution Ann Vasc Surg 2015 29 02 392 396 25433281 10.1016/j.avsg.2014.10.020

[7] Bertoglio L Salvati S Fittipaldi A Carotid to subclavian bypass and Amplatzer vascular plug subclavian endovascular occlusion before thoracic open or endovascular repair J Vasc Surg 2020 71 05 1480 14880 31630886 10.1016/j.jvs.2019.08.237

[8] Güneyli S Çinar C Bozkaya H Parıldar M Oran İ Applications of the Amplatzer Vascular Plug to various vascular lesions Diagn Interv Radiol 2014 20 02 155 159 24047719 10.5152/dir.2013.13139 PMC4463302

